# Regional binding of tau and amyloid PET tracers in Down syndrome autopsy brain tissue

**DOI:** 10.1186/s13024-020-00414-3

**Published:** 2020-11-23

**Authors:** L. Lemoine, A. Ledreux, E. J. Mufson, S. E. Perez, G. Simic, E. Doran, I. Lott, S. Carroll, K. Bharani, S. Thomas, A. Gilmore, E. D. Hamlett, A. Nordberg, A. C. Granholm

**Affiliations:** 1 Division of Clinical Geriatrics, Center for Alzheimer Research, Department of Neurobiology, Care Sciences and Society, Karolinska Institutet, Stockholm, Sweden; 2 Knoebel Institute for Healthy Aging (KIHA), University of Denver, Denver, CO USA; 3 Department of Neurobiology and Neurology, Barrow Neurological Institute, Phoenix, AZ USA; 4 Department of Neuroscience, Croatian Institute for Brain Research, University of Zagreb Medical School, Zagreb, Croatia; 5 University of California Irvine, Irvine, CA USA; 6 Department of Pathology and Laboratory Medicine, Medical University of South Carolina, Charleston, SC USA; 7 Theme Aging, Karolinska University Hospital, Stockholm, Sweden

**Keywords:** Neurofibrillary tangles, Alzheimer’s disease, PET tracers, Down syndrome, Neuropathology

## Abstract

**Introduction:**

Tau pathology is a major age-related event in Down syndrome with Alzheimer’s disease (DS-AD). Although recently, several different Tau PET tracers have been developed as biomarkers for AD, these tracers showed different binding properties in Alzheimer disease and other non-AD tauopathies. They have not been yet investigated in tissue obtained *postmortem* for DS-AD cases. Here, we evaluated the binding characteristics of two Tau PET tracers (^3^H-MK6240 and ^3^H-THK5117) and one amyloid (^3^H-PIB) ligand in the medial frontal gyrus (MFG) and hippocampus (HIPP) in tissue from adults with DS-AD and DS cases with mild cognitive impairment (MCI) compared to sporadic AD.

**Methods:**

Tau and amyloid autoradiography were performed on paraffin-embedded sections. To confirm respective ligand targets, adjacent sections were immunoreacted for phospho-Tau (AT8) and stained for amyloid staining using Amylo-Glo.

**Results:**

The two Tau tracers showed a significant correlation with each other and with AT8, suggesting that both tracers were binding to Tau deposits. ^3^H-MK6240 Tau binding correlated with AT8 immunostaining but to a lesser degree than the ^3^H-THK5117 tracer, suggesting differences in binding sites between the two Tau tracers. ^3^H-THK5117, ^3^H-MK6240 and ^3^H-PIB displayed dense laminar binding in the HIPP and MFG in adult DS brains. A regional difference in Tau binding between adult DS and AD was observed suggesting differential regional Tau deposition in adult DS compared to AD, with higher THK binding density in the MFG in adult with DS compared to AD. No significant correlation was found between ^3^H-PIB and Amylo-Glo staining in adult DS brains suggesting that the amyloid PIB tracer binds to additional sites.

**Conclusions:**

This study provides new insights into the regional binding distribution of a first-generation and a second-generation Tau tracer in limbic and neocortical regions in adults with DS, as well as regional differences in Tau binding in adult with DS vs. those with AD. These findings provide new information about the binding properties of two Tau radiotracers for the detection of Tau pathology in adults with DS in vivo and provide valuable data regarding Tau vs. amyloid binding in adult DS compared to AD.

**Supplementary Information:**

The online version contains supplementary material available at 10.1186/s13024-020-00414-3.

## Background

Down syndrome (DS) is the most prevalent genetic chromosomal abnormality characterized by a triplication of chromosome 21, where the amyloid precursor protein gene (*APP*) is located [1]. This is, in part, responsible for the increased incidence of Alzheimer’s disease (AD) in DS (DS-AD), with higher prevalence and earlier age of onset than sporadic AD [1–3]. Evaluations of autopsy cases reveal that at 35 years of age, virtually all individuals with DS display AD pathology characterized by amyloid plaques and hyperphosphorylated Tau (p-Tau) containing neurofibrillary tangles (NFTs) [2–4]. Similar to clinical findings in sporadic AD, these lesions are accompanied by early decline in verbal and visual episodic memory and verbal fluency, followed later by an impairment in informant measures [5, 6].

Even though it is more challenging to conduct positron emission tomography (PET) and magnetic resonance imaging (MRI) imaging studies on people with DS, a recent in vivo study using ^18^F-AV1451 Tau PET and ^11^C-PIB amyloid PET revealed a positive correlation between Tau deposits and amyloid burden [7]. In a small cohort of adults with DS, brain Tau deposition imaged by ^18^F-AV1451 was found to be correlated with amyloid binding and age, as well as with progressive neurodegeneration as evaluated with ^18^F-fluorodeoxyglucose (FDG)-PET and MRI [7]. Previous studies using Positron Emission Tomography (PET) imaging revealed that amyloid-beta accumulation occurs prior to dementia onset in DS [8]. However, although Tau binding cannot be found earlier than age 30 in people with DS, it is not known whether this is due to limitations of PET sensitivity or reduced binding to certain forms of pathological Tau. Overall, it has been a challenge to find Tau tracers that specifically bind to Tau in brain. In our hands, the first-generation Tau tracer THK5117 tracer demonstrated a distinct laminar binding pattern in some cortical areas in patients with AD, which corresponded with AT8 immunostaining, but there were some regional differences that potentially reflected off-target binding [9]. A second generation of Tau tracers has been developed including MK6240. Based on in silico modeling suggesting the existence of at least four binding sites on AD Tau fibrils [10] and different binding properties for the tau tracers, we utilized both ^3^H-THK5117 and ^3^H-MK6240 Tau tracers in the present study.

In the current study, we used autoradiography to visualize the binding of two Tau tracers in order to determine potential differences in distribution in two different brain regions that display AD pathology, medial frontal gyrus (MFG) and hippocampus (HIPP) between DS-AD and sporadic AD. Autoradiography with PET tracers specific for Tau and amyloid was carried out using fixed sections of post mortem tissue obtained from people with DS of different ages obtained from three different brain banks in comparison to age-matched AD and control cases. Tau immunohistochemistry (AT8 antibodies) and aggregated amyloid (Amylo-Glo) histochemistry were performed on adjacent sections to validate the specificity of the autoradiographic binding. The overall aims of the current study were: 1) to investigate the binding properties of a first-generation and a second-generation Tau tracer compared to a commonly used amyloid tracer in DS and AD tissue, and 2) to examine regional differences in Tau and amyloid binding in DS-AD and DS-MCI tissue versus early onset (EOAD) and late onset (LOAD) AD tissue.

## Material and methods

### Chemicals

^3^H-THK5117 (specific activity (SA) = 75 Ci/mmol), ^3^H-PIB: SA = 73 Ci/mmol and THK5117 from Novandi chemistry AB (Södertälje, Sweden). BTA-1 (2-(4′-Methylaminophenyl)benzothiazole) was purchased from Sigma-Aldrich, USA.) ^3^H-MK6240 and MK6240 were synthesized by Merck & Co (West Point, PA, USA; SA = 44 Ci/mmol). Immunocytochemistry used an AT8 mouse monoclonal phospho-Tau (epitope Ser202, Thr205) antibody (Invitrogen CA, USA) and Amylo-Glo ready to dilute (RTD, Biosensis, Therbarton, Australia) was used to visual amyloid.

### Human brain tissues

Formalin-fixed paraffin-embedded brain sections (5–10 μm) from DS-AD (*n* = 7), AD (*n* = 9), and control (*n* = 8) cases) were assessed. Tissues were obtained from: the Croatian Institute for Brain Research (Dr. Goran Simic), the Carroll A. Campbell Jr. Neuropathology Laboratory (CCNL) at the Medical University of South Carolina (MUSC, Dr. Steve Carroll and Dr. Eric Hamlett), and University of California, Irvine, Alzheimer’s Disease Research Center (UCI-ADRC) and the Institute for Memory Impairments and Neurological Disorders (Dr. Ira Lott and Dr. Eric Doran). This research was completed under each University’s Institutional Review Board (IRB) with either the determination of Not Human Research (NHR) Criteria set forth by the Code of Federal Regulations (45CFR46) or as Human Subjects Research with the requirement for written informed consent from the participants or the participants Legally Authorized Representative per local statute (UCI-ADRC). The fetal tissue was obtained from the Croatian Brain Bank according to NIH regulations for fetal tissues. The brain tissue processing and neuropathological staging of AD and control cases was assessed by a board-certified neuropathologist and included assessment of CERAD plaque score and Braak stage according to NIA protocols [11]. To overcome potential inter-cohort variations in brain processing procedures, tissues from all cohorts were processed simultaneously by the same investigator for each experimental condition. In addition, amyloid and tau staining were performed by the same lab for all cases and each brain region to confirm neuropathological staging for every case (see Table 1 for case demographics.)

**Table 1:** Neuropathological staging of AD was confirmed using H & E, silver, amyloid and p-Tau staining, which included Braak and CERAD stages as shown in Table 1 All EOAD cases, with the exception of one, had a Braak stage of V-VI and a CERAD plaque stage of C (definite AD). LOAD cases were Braak V-VI and a CERAD of C, except for two cases; one had a Braak stage of V-VI and a CERAD of 0-A and a second had Braak stage of VI and a CERAD of B. For the DS cases, all except two DS-MCI cases had a Braak of VI and a CERAD stage of C (definite AD). However, the two DS-MCI cases had significant accumulation of both NFTs and plaques with Braak III-IV and IV-V, respectively and both were a CERAD of C. Finally, the control cases (aged 42–100 years) had a Braak stage of 0-III with CERAD staging of 0-B. The 100-year-old case had a CERAD plaque density of C and was excluded due to the high plaque count, leaving an N of 8 in that group. Importantly, examination of the clinical records of the nine control cases revealed reports of cognitive impairment. Most of the control cases (7 out of 9) originated from the Medical University of South California (MUSC) brain bank, where a full medical history was available. The fetal DS and control tissues were obtained from University of Zagreb without information other than the gestational week, gender, and DS vs. normosomic status. The average post-mortem interval (PMI) was significantly higher (*p* = 0.019) for the control cases (5.75–25 h) compared to DS cases (one-way ANOVA: F_2,16_ = 4.729, *p* = 0.024). However, no significant differences in PMI were observed between DS-AD and AD. No correlations between PMI and outcome measures (PIB, THK5117, Amylo-glo or AT8) were found suggesting that PMI was not acting as a covariate

Group	Number/group	Female/male	Age (±SD)	Age range	PMI	Braak range	CERAD range
AD	*N =* 9	7F/2M	71 ± 11.67	55–85	10.44 (6.5–17.25)	II-VI	C
DS-AD	*N =* 7	6F/1M	54 ± 7	42–62	6.4 (2.4–18.4)	III-VI	C
CN	*N =* 8	2F-6M	63 ± 14	42–88	16.6 (5.75–25)	I-IV	0-B
Fetal DS	*N =* 2	1F/1M		20-23gw	Not available	0	0
Fetal CN	*N =* 3	2F/1M		20-23gw	Not available	0	0

### Autoradiography

Autoradiography was performed at Karolinska Institute using 5–10 μm thick paraffin sections from the MFG and HIPP from DS-AD, AD and age-matched control cases. Autoradiography was performed at room temperature (RT) after deparaffinization. For ^3^H-PIB, the sections were pre-incubated (15 min) with binding buffer (phosphate-buffered saline (PBS) + 1% BSA) and then 45 min with 1 nM of ^3^H-PIB in binding buffer + 10% ethanol. Non-specific binding (NSP) was determined by incubation with 10 μM of unlabeled BTA-1. For ^3^H-THK5117 and ^3^H-MK6240 sections were pre-incubated for 15 min in PBS containing 0.1% BSA followed by a 1-h incubation with ^3^H-THK5117 (3–4 nM) or ^3^H-MK6240 (1 nM). NSP was assessed by incubation with unlabeled THK5117 (10 μM) or unlabeled MK6240 (1 μM). Binding was terminated by washing 3X5 min with cold binding buffer and distilled water. Sections were dried and apposed for 4 days for ^3^H-THK5117 and 7 days for ^3^H-PiB and ^3^H-MK6240 on a phosphoplate and read using a BAS-2500 imager (BAS-TR2040, Fuji imaging plate, Fujifilm, Tokyo, Japan) and analyzed using multigauge software, Fujifilm, Tokyo, Japan). Non-specific binding was subtracted from total binding to obtain specific binding densities for each tracer and brain region.

### Immunostaining

To confirm that the Tau tracers were binding to regions containing Tau, AT8 immunostaining was performed on adjacent sections at Barrow Neurological Institute. Paraffin-embedded sections were deparaffinized and pre-treated for antigen retrieval using formic acid, as previously described [12]. Endogenous peroxidase activity was blocked by 1% hydrogen peroxide, followed by incubation overnight with the AT8 monoclonal antibody (1:1000, Invitrogen). After washing, sections were incubated with biotin-conjugated secondary antibodies, washed in PBS, incubated with streptavidin-horseradish peroxidase (HRP) complex and visualized using a 1 mg/ml diaminobenzidine (DAB) solution containing 0.02% H_2_O_2_, dehydrated and cover-slipped. Immunostained controls include deletion of the primary or secondary antibodies.

To confirm that the PIB binding was targeting amyloid, amyloid plaque staining was completed at University of Denver (DU) using Amylo-Glo RTD Staining reagent (Biosensis #TR-300-AG) following the manufacturer’s protocol. Briefly, rehydrated sections were placed in 70% ethanol, rinsed in distilled water, incubated in the Amylo-Glo RTD solution, rinsed in 0.9% saline solution for 5 min, briefly washed in distilled water and cover slipped with ProLong Diamond Antifade. Amyloid plaques labeled with Biosensis’ Amylo-Glo RTD were visualized using UV epifluorescent illumination attached to a Nikon Optiphot microscope (Eclipse 600). Each staining batch contained sections from all groups to avoid potential variability resulting from different processing protocols at the different brain banks.

### Semi-quantitative data analysis

For autoradiography, the gray matter was used as a region of interest (ROI) and drawn manually both on the total and the non-specific autoradiogram using Multi Gauge V3.0 software (Fujifilm, Tokyo, Japan) [13]. Values were transformed from PSL/mm^2^ into fmol/mg using a tritium Standard and specific binding was determined by subtracting the non-specific binding from total binding. For the AT8 and Amylo-Glo stains, densitometric measures were obtained using the Image J density plugin, with a grayscale of 0–256 and background subtraction [14]. Three arbitrary frames were placed in the gray matter in each target area, mean density was obtained and background subtracted from a non-stained portion of each section within the ROI. An average density was obtained in each section and a mean value per brain region determined.

### Statistical methods

Statistical analysis was performed using GraphPad Prism, version 6 (GraphPrism Software, La Jolla, CA, USA). Checking for outliers in each data set using Grubb’s test resulted in one outlier excluded from the Control group for Amylo-Glo staining density. One-way ANOVA followed by Tukey’s multiple comparisons test was used for the comparison between groups (AD, DS-AD, control) and regions (MFG and HIPP) for PET tracer binding. Significance was set at *p* < 0.05. Pearson correlations were used to assess associations between ^3^H-THK5117, ^3^H-MK6240 and AT8, and between ^3^H-PiB and Amylo-Glo.

## Results

### Autoradiography

**Fig. 1:**
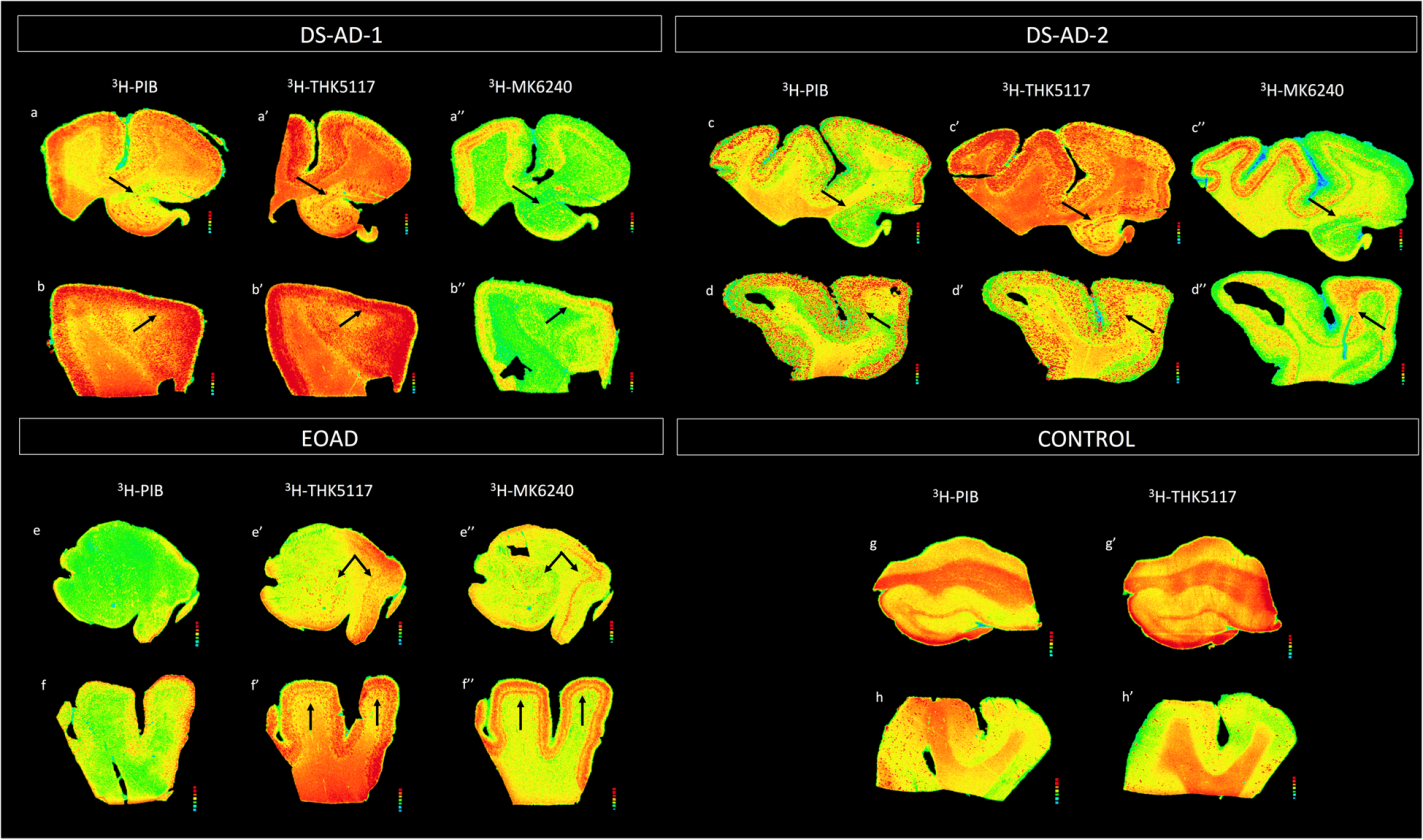
Amyloid and Tau autoradiography binding in 2 DS-AD in comparison to 1 EOAD and 1 control. Top row: DS-AD-1 57-year-old female with DS-AD. From left to right: Autoradiographic images showing ^3^H-PIB binding (a, b),^3^H-THK5117 binding (a’,b’) and ^3^H-MK6240 binding (a”,b”) on an adjacent section in HIPP and MFG. DS-AD-2 62-year-old female with DS-AD From left to right: Autoradiographic images showing ^3^H-PIB binding (c,d),^3^H-THK5117 binding (c’,d’) and ^3^H-MK6240 binding (c”,d”) on an adjacent section in HIPP and MFG. Bottom row: EOAD: 55-year-old female diagnosed with Alzheimer’s disease: From left to right: Autoradiographic images showing ^3^H-PIB binding (e,f),^3^H-THK5117 binding (e’,f’) and ^3^H-MK6240 binding (e”,f”) on an adjacent section in HIPP and MFG. CONTROL: 59-year-old control case. From left to right: Autoradiographic images showing ^3^H-PIB binding (g, h),^3^H-THK5117 binding (g’,h’) on an adjacent section in HIPP and MFG

Figure 1 illustrates the overall distribution of total binding of ^3^H-PiB, ^3^H-THK5117, and ^3^H-MK6240 tracers in HIPP and MFG of 2 DS-AD cases, 1 EOAD case and 1 control. The binding patterns were dense in HIPP and MFG in DS-AD for the three tracers. Although ^3^H-PIB binding was mainly limited to the molecular layer of the dentate gyrus (DG) and CA2/CA1 pyramidal cell layers, some ^3^H-PiB positive binding was observed in the hilar region of the HIPP (Fig. 1a, c). By comparison, hippocampal ^3^H-THK5117 binding was more intense and widespread within the hilar region (Fig. 1a’, c’). Specific binding was observed in both inner and outer molecular layers of the DG, in the pyramidal cell layer and in the stratum oriens in CA3 and CA1 (Fig. 1a’,c’). ^3^H-MK6240 labeling revealed similar laminar binding patterns to ^3^H-THK5117 in the HIPP (Fig. 1a”, c”), albeit with less intense labeling. ^3^H-MK6240 binding appeared more specific with less non-specific binding in the white matter. This was also observed in MFG (Fig. 1b’,d’). Although intense ^3^H-PIB binding was found throughout all cortical layers of the MFG in DS-AD cases (Fig. 1b, d), ^3^H-THK5117 binding exhibited a laminar specific increase in layers II-III and V-VI (Fig. 1b’,d’). Less ^3^H-THK5117 binding was observed in layer I in MFG (Fig. 1b’,d’).

Layer-specific binding was observed in the MFG for ^3^H-THK5117 (Fig. 1f’) and was even more pronounced for ^3^H-MK6240 (Fig. 1f”). ^3^H-THK5117 binding was widespread throughout the hippocampal formation in EOAD (Fig. 1e’), while a strong band-like distribution of Tau binding in the MFG was seen for both ^3^H-THK5117 and ^3^H-MK6240 in AD (Fig. 1f, f’). Very little, if any specific binding, was observed in HIPP (Fig. 1g, g’) and MFG (Fig. 1h, h’) for ^3^H-PiB and ^3^H-THK5117 in the age-matched control cases. ^3^H-PIB and ^3^H-THK5117 autoradiographic binding in the hippocampus of LOAD is presented in supplemental Fig. 1.

### Histochemical staining

**Fig. 2:**
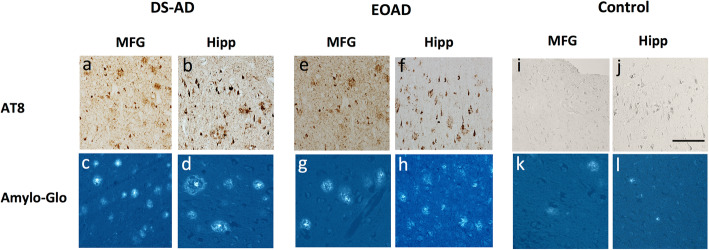
Amylo-Glo and AT8 immunostaining in HIPP and MFG of 1 DS-AD, 1 EOAD and 1 control case. From left to right: AT8 staining in DS-AD (a,b) and Amylo-Glo staining (c,d) in MFG and HIPP respectively. AT8 staining in 1 EOAD (e,f) and Amylo-Glo staining (g,h) in MFG and HIPP respectively. AT8 staining in 1 control (i,j) and Amylo-Glo staining (k,l) in MFG and HIPP respectively

To confirm tracer binding to amyloid and Tau, we performed staining for each of these markers in adjacent sections. Staining patterns for these markers suggested that the autoradiographic binding corresponded to amyloid and p-Tau staining in DS-AD, EOAD, LOAD and control cases. AT8 immunostaining observed in DS-AD and AD suggested that the Tau immunostaining, at least in the forms that are recognized by the AT8 antibody (phosphorylated Tau at serine 202 and threonine 205) displayed a similar regional distribution to that found with ^3^H-THK5117 and ^3^H-MK6240. AT8 positive NFTs, ghost tangles and neurites were observed throughout the CA1 pyramidal cell layer, with frequent AT8-reactive neurites near plaques (Fig. 2, supplementary Fig. 1).

### Quantification

**Fig. 3:**
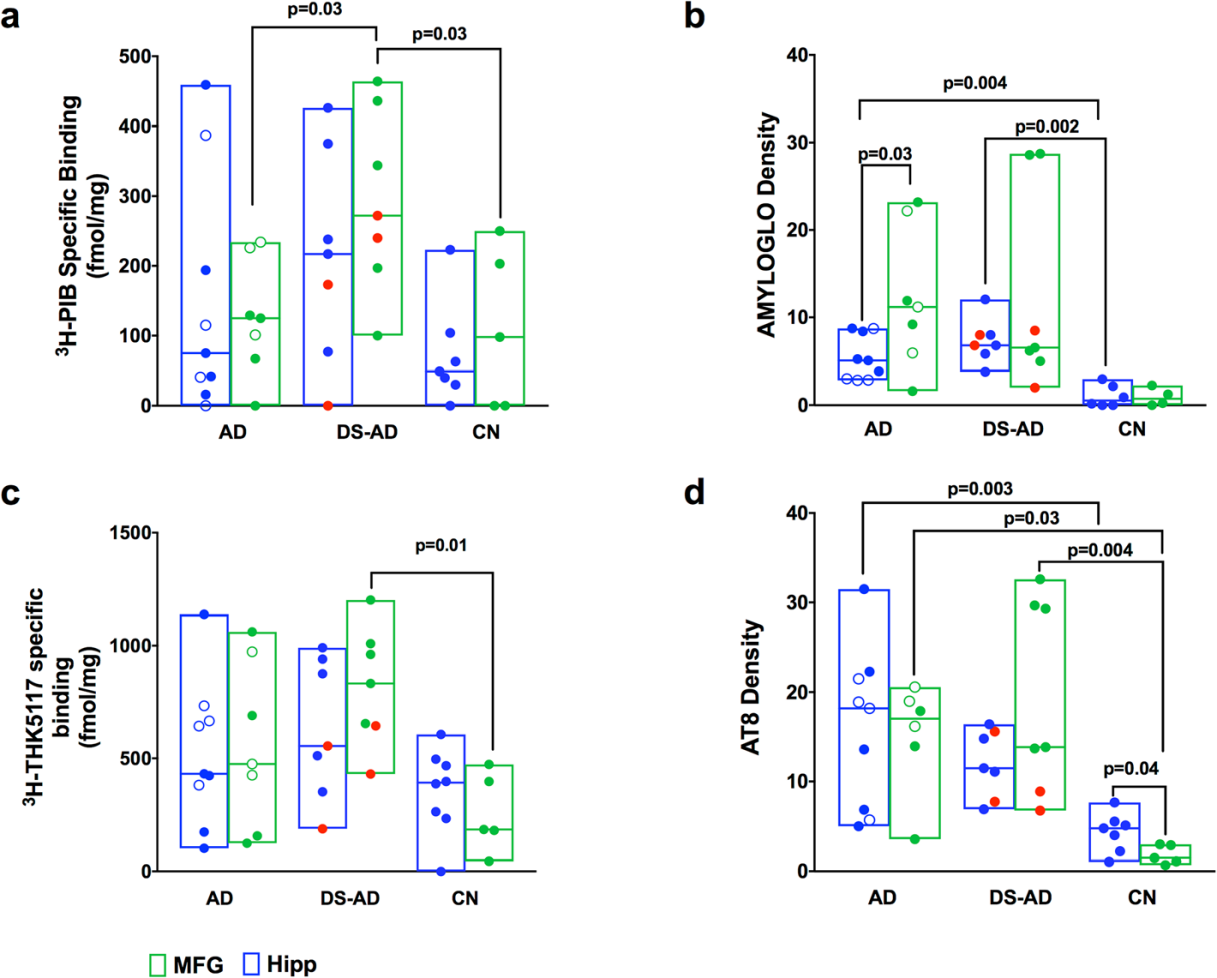
Semi-quantitative analysis of ^3^H-PiB, ^3^H-THK5117 autoradiography and Amylo-Glo and AT8 staining. Box plot with a line at the median represent: **a**: Specific binding of ^3^H-PIB and for AD, DS and CN in HIPP and MFG. **b**: Amylo-Glo density for AD, DS and CN in HIPP and MFG. **c**: Specific binding of ^3^H-THK5117 for AD, DS and CN in HIPP and MFG. **d**: AT8 density for AD, DS and CN in HIPP and MFG. Specific binding for ^3^H-PIB and ^3^H-THK5117 is represented in fmol/mg. Gray matter was delineated manually using multigauge software. Density values for Amylo-Glo and AT8 are presented in b and d respectively. The red dots in each plot represent two DS-MCI cases. The empty blue circles in AD represent the EOAD

**Fig. 4:**
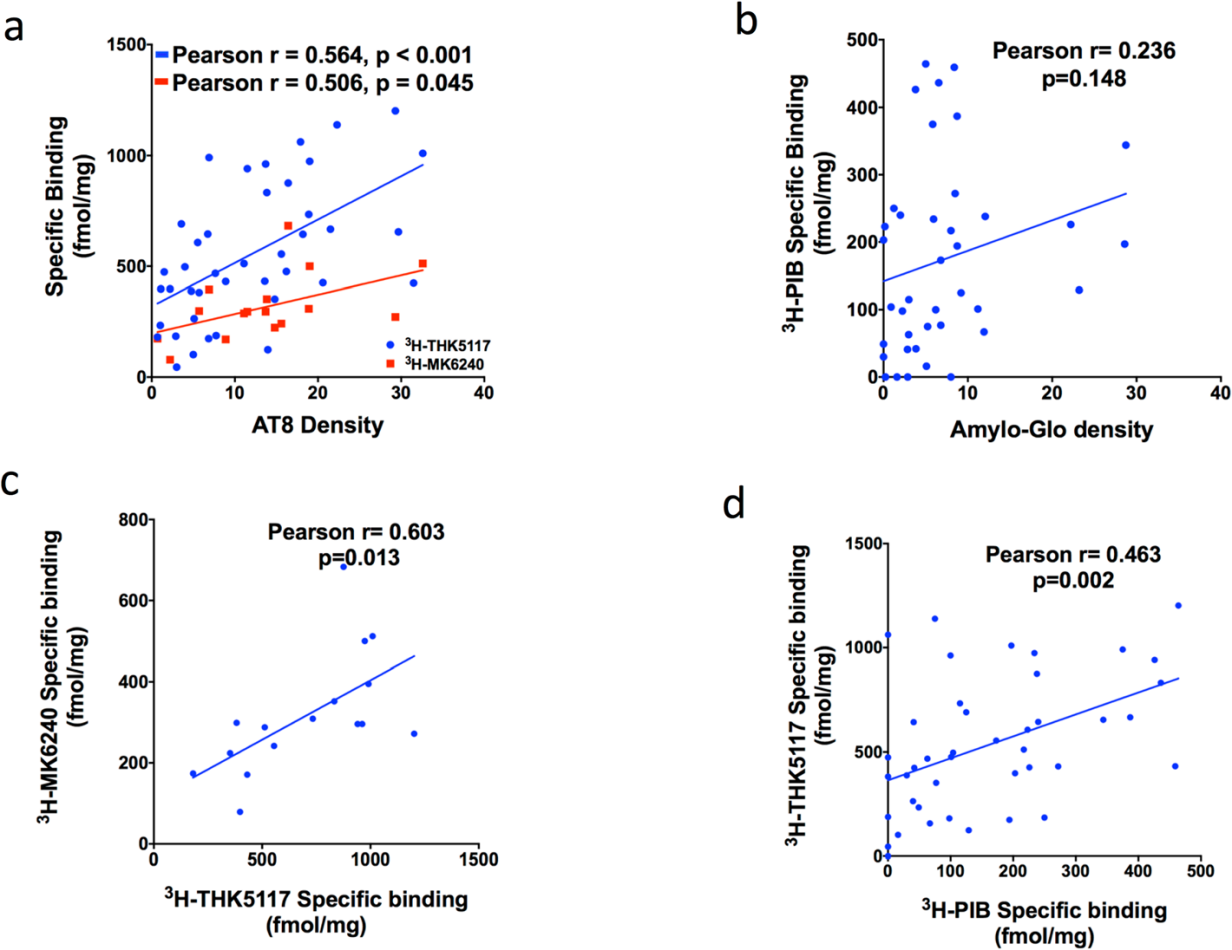
Linear regressions between autoradiography binding and density staining. **a**. ^3^H-THK5117 and AT8 immunostaining (Pearson *r =* 0.564, *p* < 0.001, shown in blue), and ^3^H-MK6240 and AT8 immunostaining (Pearson *r =* 0.506, *p* = 0.045, shown in red) in the whole cohort (AD, DS-AD, CN) in both regions (MFG + HIPP). **b**: ^3^H-PIB and Amylo-Glo (Pearson *r =* 0.236, *p* = 0.148) in the whole population (AD, DS-AD,CN) in both regions (MFG + HIPP). **c**: ^3^H-THK5117 and ^3^H-MK6240 (Pearson *r =* 0.603, *p* = 0.013) in the whole population (AD, DS-AD,CN) in both regions (MFG + HIPP). **d**: Correlation in the whole population (AD,DS-AD,CN) in both regions (MFG + HIPP) between ^3^H-PIB and ^3^H-THK5117(Pearson *r =* 0.463, *p* = 0.002)

The density measurements for the EOAD (*n* = 4) and LOAD (*n =* 4) cases were pooled and compared with the DS-AD cases in order to be able to performed statistical calculations. In addition, two available DS-MCI cases were included in the DS-AD group. Quantification of autoradiographic specific binding, as well as corresponding immunostaining are shown in Fig. 3. ^3^H-THK5117 was significantly different in the three groups in the MFG (F_2, 16_ = 5.489, *p* = 0.015) with higher specific binding in DS-AD in comparison to controls (*p* = 0.012) (Fig. 4c). For ^3^H-PIB, a significant difference was observed between the three groups in MFG (F_2, 16_ = 5.479, *p =* 0.015) with higher specific binding observed in DS-AD in comparison to AD (*p =* 0.031) and controls (*p =* 0.031) (Fig. 3a). No differences across groups were observed in HIPP for ^3^H-PIB (Fig. 3a) or ^3^H-THK5117 (Fig. 3c).

No significant differences in Amylo-Glo staining between groups (DS-AD, AD, and controls) were observed in the MFG (Fig. 3b). Significant differences were found between the three groups in AT8 staining (F_2, 15_ = 7.677, *p =* 0.005), with both the AD (*p =* 0.031) and DS-AD (*p* = 0.004) groups exhibiting significantly higher density compared to the MFG control group (Fig. 3d).

In the HIPP, Amylo-Glo density showed significant differences between the three groups (F_2, 19_ = 12.8, *p* = 0.0003) with higher binding in AD (*p =* 0.005) and DS-AD (*p* = 0.0002) compared to controls (Fig. 3b). For AT8 staining, we observed a significant difference between the three HIPP groups (F_2, 20_ = 7.133, *p =* 0.005) with a significant difference between AD and controls (*p* = 0.003) (Fig. 3d). Finally, a comparison between the two brain regions (unpaired Student’s t-test) for each tracer and stain revealed significantly higher Amylo-Glo staining in MFG compared to HIPP only for AD (*p =* 0.03, Fig. 3b). We also found that AT8 staining was significantly greater in HIPP compared to MFG and control groups (*p =* 0.042, Fig. 3d). Although this study included only a few LOAD (*n* = 5 for HIPP and *n* = 4 for MFG) and a few EOAD cases (*n =* 4 for HIPP and *n* = 3 for MFG), we could observe some differences between EOAD and LOAD in HIPP for THK5117 and in MFG for PIB (see supplemental Fig. 2). DS-AD showed higher binding than EOAD and LOAD in MFG for both ^3^H-THK5117 and ^3^H-PIB (see supplemental Fig. 2). DS-MCI cases showed somehow lower density of amyloid and Tau tracers compared to their DS-AD counterparts, although only two cases of DS-MCI were available for the study.

Fig. 4 shows the association between the ^3^H-PIB specific binding densities and Amylo-Glo staining (Fig. 4b) as well as between ^3^H-THK5117 and ^3^H-MK6240, and AT8 immunostaining (Fig. 4a). Although no relationship was observed between ^3^H-PIB and Amylo-Glo staining (Fig. 4b), there was a significant positive correlation between ^3^H-THK5117, ^3^H-MK6240 and AT8 immunostaining. A significant correlation was also observed between THK5117 and MK6240 (Fig. 4c).

Similar to in vivo PET imaging, we found a significant positive relationship between ^3^H-THK5117 and ^3^H-PIB binding (Fig. 4d), showing that ex vivo autoradiography corresponds to findings observed in in vivo AD cases. Tau and amyloid did not correlate in cases with more pronounced AD pathology.

### ^3^H-THK5117 and ^3^H-MK6420 binding in fetal DS and control cases

Total autoradiographic binding from a fetal control and a fetal DS case are presented in supplementary Fig. 3. We observed that ^3^H-THK5117 and ^3^H-MK6240 binding was evident in the outer and inner layers of the developing fetal DS cortex. No specific Tau binding was observed in the control fetal cases examined. These preliminary observations warrant further examination of Tau binding on a larger cohort, both in fetuses and children with DS to explore potential early Tau alterations in DS.

## Discussion

The current study examined the laminar distribution of two different Tau and one amyloid tracer in two brain regions obtained *post-mortem* from DS-AD, AD and control cases. The AD cases included equal numbers of EOAD and LOAD cases (Fig. 3). Indeed, to compare the DS-AD group with sporadic AD group we decided to include both EOAD and LOAD as well as age matched control. We observed binding with ^3^H-THK5117, ^3^H-MK6240 and ^3^H-PIB binding was observed in the MFG and HIPP in all DS-AD cases. There was a significant positive correlation between AT8 immunostaining and ^3^H-THK5117 autoradiography when all samples and brain regions were pooled. Although ^3^H-THK5117 has been shown to display off-target binding to monoamine oxidase B (MAO-B, [15]), the enzyme was probably denatured by heat during the paraffin embedding process [16]. DS-AD cases exhibited significantly higher ^3^H-THK5117 binding in the MFG than controls. Interestingly, the two DS-MCI cases (labeled red in Fig. 3) exhibited lower Tau but not amyloid binding compared to DS-AD cases (*n* = 5) in the MFG, suggesting that Tau activity increases incrementally in this brain region in adult with DS during the transition from MCI to AD. This concept is supported by our previous studies using exosome biomarkers [17] and warrants further study including nondemented adults with DS in a larger cohort of cases. Significantly higher binding of ^3^H-PIB was also seen in the MFG of DS-AD compared to AD and EOAD cases exhibited higher ^3^H-PIB binding than the LOAD cases in the MFG. Despite the increased ^3^H-PIB binding and Amylo-Glo staining in the MFG in EOAD compared to LOAD, DS-AD cases exhibited higher PIB binding than EOAD. Tissue labeled with the Tau tracer ^3^H-MK6240, revealed a similar but weaker regional distribution-binding pattern in the MFG. Furthermore, autoradiograms revealed distinct bilaminar cortical ^3^H-THK5117 binding in fetal DS cases compared to controls (Supplemental Fig. 3). Tau tracer binding does not necessarily indicate the presence of pathological Tau deposits. Abnormal Tau deposits may be related to brain connectional and neuronal development [18]. Further studies of gestational DS tissue are needed to better understand the functional consequences of cortical fetal tau. A recent immunohistochemical and western blot study showed a loss of normal Tau phosphorylation in fetal DS tissue, although phosphorylation-independent Tau staining did not change, suggesting complicated Tau biology early in development [19]. Since the binding characteristics of the two Tau tracers used herein have not been examined in fetal tissue to date, it is not known whether the binding observed here represents total Tau or a phosphorylated form. Although this was not the focus of the current study, the findings are interesting and warrant future in-depth investigation. PiB binding or Amylo-Glo staining was not found in fetal DS or control tissue, suggesting a lack of aggregated amyloid at the gestational weeks examined, consistent with earlier studies [20]. Since the data generated from the present Tau ligand investigations reported showed a similar distribution to that seen with AT8 immunostaining in adult brain suggesting that the Tau binding is, in part, associated with pathological events in DS. On the other hand, despite the correspondence between AT8 immunostaining and ^3^H-THK5117 and ^3^H-MK6240 binding patterns in DS and AD, it is possible that the binding patterns the two Tau PET tracers label other forms of Tau or other aggregating proteins.

We did not find a correlation between Amylo-Glo staining and ^3^H-PIB binding in any of the groups examined, suggesting the existence of different binding targets for these two markers. For example, although [^11^C]-PIB used in PET imaging, has specificity for fibrillar amyloid [21] it also binds to lower assemblies like protofibrils [22] and to a lesser degree to NFTs [23, 24], in comparison to Amylo-Glo, which only stains amyloid plaques [25]. Although a limitation of ^3^H-PiB autoradiography is high non-specific binding [23], others have shown a strong correlation between increased Aβ immunostaining in *postmort*em tissue and higher [^11^C]-PIB uptake using PET imaging [26]. Here, we found significantly higher specific binding for [^3^H]-PIB in the MFG in DS-AD compared to AD and control cases. Interestingly, others report that the striatum is the first area to exhibit detectable PIB binding in DS-AD [6] suggesting that it would be interesting to include this region in further Tau binding studies. In the present study, amyloid and tau binding correlated with each other, but whether this occurs in advanced AD where amyloid load likely plateaus [26] and Tau is proposed to increase with greater loss of cognition remains an under investigated question.

Early impairment in neuronal differentiation leads to reduce gray matter volume, especially in the frontal cortex of people with DS [27]. Our findings are consistent with earlier findings as densities of both [^11^C]-PIB and ^3^H-THK5117 binding were slightly higher in the MFG than in the HIPP in DS-AD cases. Imaging and pathological studies have suggested that the frontal cortex is an early area of AD pathology in DS [27, 28]. Therefore, our findings and others suggest that frontal cortex-related AD pathology underlies functional impairments, including reduced executive function and attention deficits, observed early in adults with DS [29] confirmed by imaging studies [6, 28] suggesting that this cortical region is an early pathological site for DS [1].

We compared the binding patterns of THK5117 with the second-generation Tau tracer ^3^H-MK6240 to validate our data since some first-generation Tau PET tracers display off-target binding to MAO A and B [15]. Despite the small sample size examined here, we observed similar binding distributions, but with a lower intensity with the MK6240 tracer. It has been shown that there are four different high-affinity binding sites on Tau fibrils [10]. Available first- and second-generation Tau tracers show varying binding affinity to different Tau binding sites. In silico modeling predicts that the THK5317 tracer binds to site 1 and 3, while the MK6240 tracer is predicted to have higher binding affinities for site 1 on fibril Tau [10]. Although two binding sites for ^3^H-THK5117 as well as ^3^H-THK5351 were observed using a Tau-rich human brain homogenates binding assay [9, 13], others have found only a single binding site for ^3^H-MK6240 [30]. These different tracer binding site affinities may underlie differences in the intensity of specific binding. It is important to note that one binding site might be more prominent at a different stage of brain development, disease or a particular clinical phenotype. Therefore, continued ex vivo characterization and comparison between the two generations of Tau tracers is of great importance, even though specific information regarding respective binding sites needs to be defined at this time. In a recent in vivo study [31], ^18^F-MK6240 binding patterns in target regions of people with AD were consistent with neuropathological neurofibrillary Tau staging with minimal off-target binding, suggesting that this second-generation Tau tracer has promise to effectively monitor Tau deposition in people with DS-AD. Since no studies have to date used MK6240 or the THK5117 to examine the distribution and intensity of binding in tissue from adults with DS in comparison to AD, the present findings are timely and highly relevant to the Tau DS and AD fields.

## Conclusions

In the adult population, both Amyloid and Tau deposits, observed with the ^3^H-PIB and ^3^H-THK5117 are higher in the MFG and to a lesser degree in the HIPP of people with DS-AD compared to age-matched controls. We found a significant increase in ^3^H-PIB in the MFG in DS-AD compared to AD, suggesting an earlier involvement of the cortex prior to the HIPP in DS vs. AD. Comparison of first- and a second-generation Tau tracer suggested potential different binding patterns at least in some cortical layers, providing important new data regarding their translation to clinic. Further, our findings showed that both PIB and THK5117 binding in the MFG was significantly higher in the DS-AD tissue compared to the control, lower in AD cases, suggesting that amyloid and Tau pathology is more prevalent in DS-AD frontal cortex, at least at the stages investigated herein. Moreover, Amylo-Glo staining displayed a significantly higher amyloid density in the Hipp in AD and DS-AD compared to controls, which was not observed for ^3^H-PIB. Finally, a caveat of this study is the relatively small sample size, which is a concern due to the relative lack of DS tissue from clinically well characterized cohorts [1] . A larger DS cohort will provide tissue for more extensive clinical pathological investigations to better understand the binding characteristic and biomarker potential for of Tau PET tracers and their relationship with amyloid ligand in both AD and DS-AD.

## Supplementary Information

**Additional file 1: Supplemental Fig. 1**: ^3^H-PIB/Amylo-Glo and ^3^H-THK5117/AT8 distribution in hippocampus of 1 LOAD a: 3H-PIB autoradiography, b:3H-THK5117 Autoradiography, c: Amylo-Glo, d:AT8.

**Additional file 2: Supplemental Fig. 2**: Semi-quantitative analyses of ^3^H-PIB, ^3^H-THK5117 autoradiography. Box and whiskers plot showing all data point represent: a: Specific binding of ^3^H-THK5117 in EOAD, LOAD, DS-AD, CN AD, in HIPP and MFG. b: Specific binding of ^3^H-PIB in EOAD, LOAD, DS-AD, CN AD, in HIPP and MFG. Specific binding for ^3^H-PIB and ^3^H-THK5117 is represented in fmol/mg. Gray matter was delineated manually using multigauge software.

**Additional file 3: Supplemental Fig. 3**: Tau binding in fetal cases of 1 DS and 1 non-DS control. Autoradiography using ^3^H-THK5117 and ^3^H-MK6240 autoradiography binding in one fetal-DS and one control fetal case. Fetuses were collected in gestational week 20. Groups were DS fetal cases (*n* = 2, age 22-23gw). Control case fetuses were collected in gestational week 20 (group fetal control cases (*n* = 3 age 20-23gw).

## Data Availability

The raw data/autoradiography/staining are available from the corresponding authors upon reasonable request.

## Abbreviations

DS: Down syndrome
APP: Amyloid precursor protein gene
AD: Alzheimer disease
DS-AD: Down syndrome-related Alzheimer’s disease
p-Tau: Hyperphosphorylated tau
NFT: Neurofibrillary tangles
NDEs: Neuron-derived extracellular vesicles
PIB: Pittsburg compound B
PET: Positron emission tomography
MFG: Middle frontal gyrus
HIPP: Hippocampus
