# Supplementary figures and images for: Regional binding of tau and amyloid PET tracers in Down syndrome autopsy brain tissue
Source: Mol Neurodegener. 2020 Nov 23;15:68. doi: 10.1186/s13024-020-00414-3 (PMC7682014; doi:10.1186/s13024-020-00414-3)

LOAD

$^3\text{H}$ -PIB

$^3\text{H}$ -THK5117

a

b

HIPP

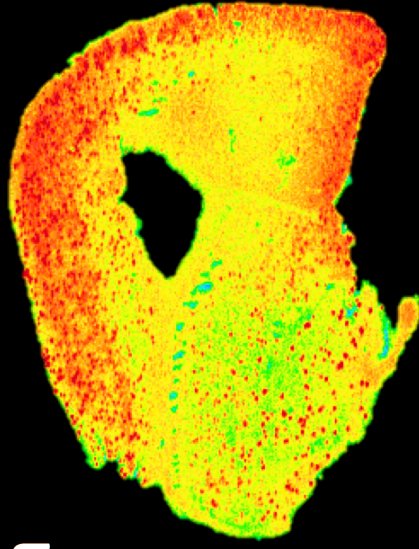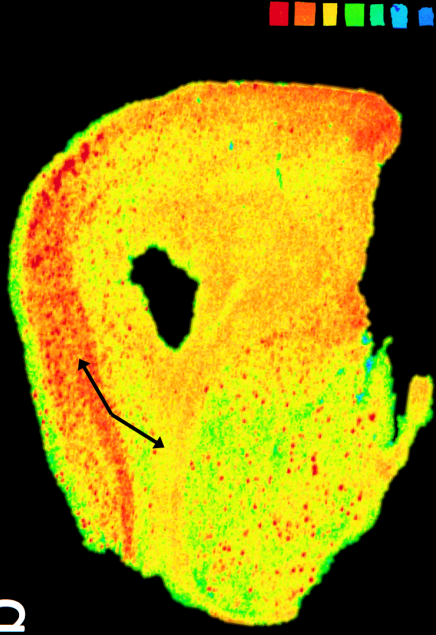

Amylo-Glo

AT8

c

d

HIPP

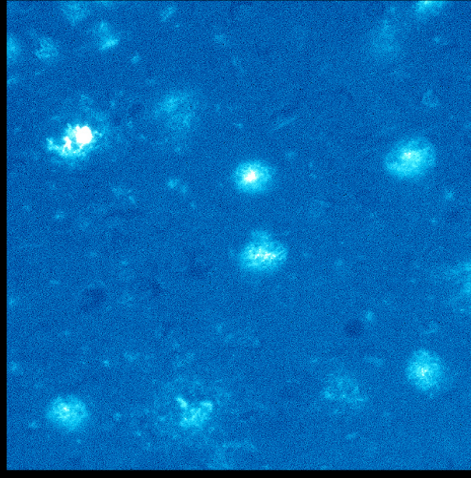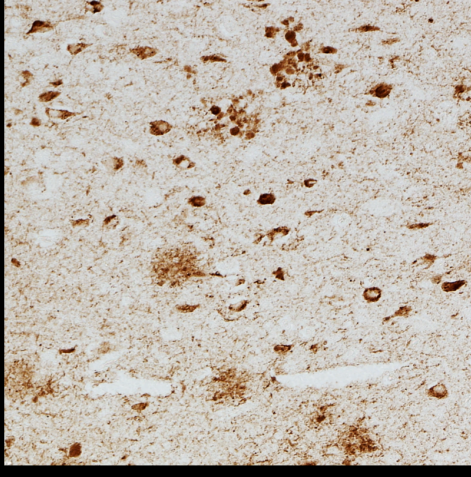

Supplement: Supplementary file 1 — Additional file 1: Supplemental Fig. 1: 3H-PIB/Amylo-Glo and 3H-THK5117/AT8 distribution in hippocampus of 1 LOAD a: 3H-PIB autoradiography, b:3H-THK5117 Autoradiography, c: Amylo-Glo, d:AT8. [file 13024_2020_414_MOESM1_ESM.pdf]

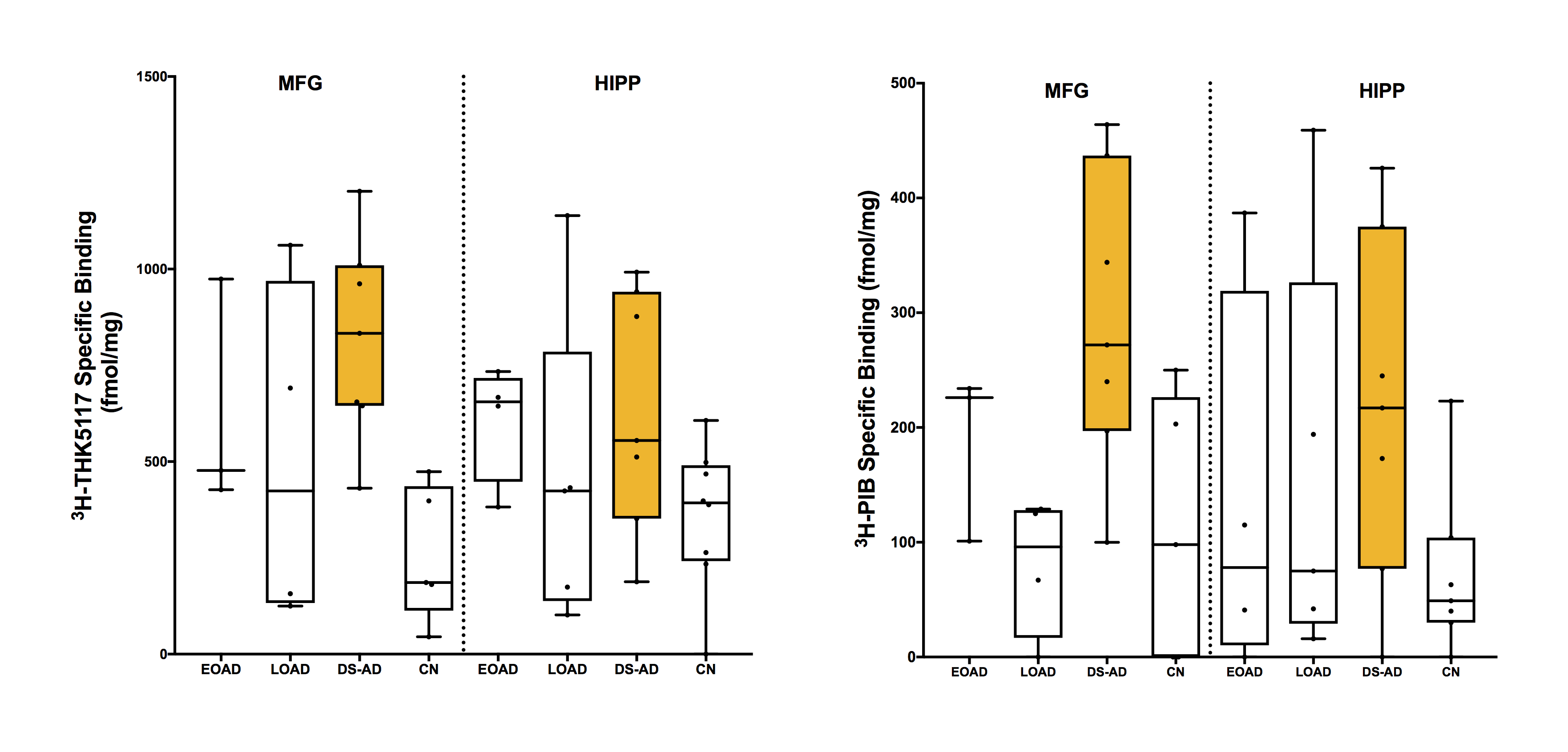

Supplement: Supplementary file 2 — Additional file 2: Supplemental Fig. 2: Semi-quantitative analyses of 3H-PIB, 3H-THK5117 autoradiography. Box and whiskers plot showing all data point represent: a: Specific binding of 3H-THK5117 in EOAD, LOAD, DS-AD, CN AD, in HIPP and MFG. b: Specific binding of 3H-PIB in EOAD, LOAD, DS-AD, CN AD, in HIPP and MFG. Specific binding for 3H-PIB and 3H-THK5117 is represented in fmol/mg. Gray matter was delineated manually using multigauge software. [file 13024_2020_414_MOESM2_ESM.tiff]

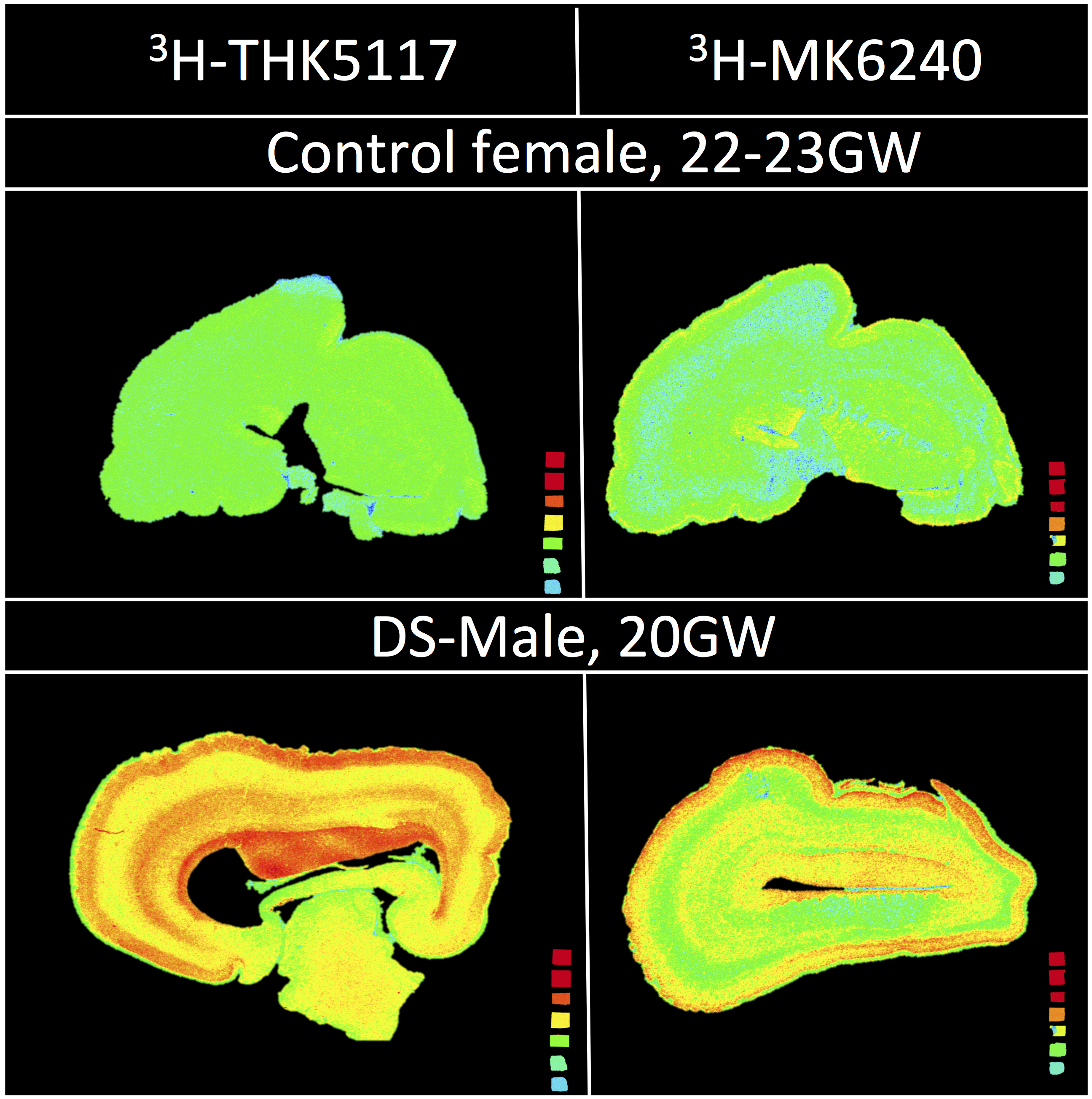

Supplement: Supplementary file 3 — Additional file 3: Supplemental Fig. 3: Tau binding in fetal cases of 1 DS and 1 non-DS control. Autoradiography using 3H-THK5117 and 3H-MK6240 autoradiography binding in one fetal-DS and one control fetal case. Fetuses were collected in gestational week 20. Groups were DS fetal cases (n = 2, age 22-23gw). Control case fetuses were collected in gestational week 20 (group fetal control cases (n = 3 age 20-23gw). [file 13024_2020_414_MOESM3_ESM.tiff]
